# Machine learning methods in sport injury prediction and prevention: a systematic review

**DOI:** 10.1186/s40634-021-00346-x

**Published:** 2021-04-14

**Authors:** Hans Van Eetvelde, Luciana D. Mendonça, Christophe Ley, Romain Seil, Thomas Tischer

**Affiliations:** 1 Department of Applied Mathematics, Computer Science and Statistics, Ghent University, Krijgslaan 281-S9, 9000 Ghent, Belgium; 2 Graduate Program in Rehabilitation and Functional Performance, Universidade Federal Dos Vales Do Jequitinhonha E Mucuri (UFVJM), Diamantina, Minas Gerais Brazil; 3 Department of Physical Therapy and Motor Rehabilitation, Faculty of Medicine and Health Sciences, Ghent University, Ghent, Belgium; 4 Ministry of Education of Brazil, CAPES Foundation, Brasília, Distrito Federal Brazil; 5 Department of Orthopaedic Surgery, Centre Hospitalier Luxembourg and Luxembourg Institute of Health, Luxembourg, Luxembourg; 6 Department of Orthopaedic Surgery, University of Rostock, Rostock, Germany

**Keywords:** Machine Learning, Injury prediction, Injury prevention, Sport injury

## Abstract

**Purpose:**

Injuries are common in sports and can have significant physical, psychological and financial consequences. Machine learning (ML) methods could be used to improve injury prediction and allow proper approaches to injury prevention. The aim of our study was therefore to perform a systematic review of ML methods in sport injury prediction and prevention.

**Methods:**

A search of the PubMed database was performed on March 24th 2020. Eligible articles included original studies investigating the role of ML for sport injury prediction and prevention. Two independent reviewers screened articles, assessed eligibility, risk of bias and extracted data. Methodological quality and risk of bias were determined by the Newcastle–Ottawa Scale. Study quality was evaluated using the GRADE working group methodology.

**Results:**

Eleven out of 249 studies met inclusion/exclusion criteria. Different ML methods were used (tree-based ensemble methods (*n* = 9), Support Vector Machines (*n* = 4), Artificial Neural Networks (*n* = 2)). The classification methods were facilitated by preprocessing steps (*n* = 5) and optimized using over- and undersampling methods (*n* = 6), hyperparameter tuning (*n* = 4), feature selection (*n* = 3) and dimensionality reduction (*n* = 1). Injury predictive performance ranged from poor (Accuracy = 52%, AUC = 0.52) to strong (AUC = 0.87, f1-score = 85%).

**Conclusions:**

Current ML methods can be used to identify athletes at high injury risk and be helpful to detect the most important injury risk factors. Methodological quality of the analyses was sufficient in general, but could be further improved. More effort should be put in the interpretation of the ML models.

**Supplementary Information:**

The online version contains supplementary material available at 10.1186/s40634-021-00346-x.

## Background

Injuries are common in individual and team sports and can have significant physical, psychosocial and financial consequences [3, 13, 22]. Understanding injury risk factors and their interplay is thereby a key component of preventing future injuries in sport [4]. An abundance of research has attempted to identify injury risk factors [4, 6, 28]. However, sports injuries are a consequence of complex interactions of multiple risk factors and inciting events making a comprehensive model necessary [6, 28]. It has to account for the events leading to the injury situation, as well as to include a description of body and joint biomechanics at the time of injury [4]. Due to the many interactions between intrinsic and extrinsic risk factors as well as their sometimes highly unpredictable nature (e.g., contact with another player), the ability to foresee the occurrence of an inciting injury event is challenging. Therefore, predictive modelling should not only focus on the prediction of the occurrence of an injury itself but, moreover, it should try to identify injury risk at an individual level and implement interventions to mitigate the level of risk [28]. In order to plan effective preventive intervention, it is therefore important to be aware both of the various isolated risk factors and their interaction [6].

In recent years, the use of advanced Artificial Intelligence (AI) methods has appeared in sports medicine to tackle this challenging multi-faceted task [1, 5, 14, 16]. AI methods have already been used successfully in sports science within the realm of game analysis, tactics, performance analysis and outcome predictions [12, 17, 21] and are about to start transforming clinical medicine [9, 31, 33, 39, 42]. However, for clinicians, the application and the understanding of AI is often difficult [24]. Therefore, the explanations of the core terms for AI application are provided in Supplementary File S1.

**Fig. 1:**
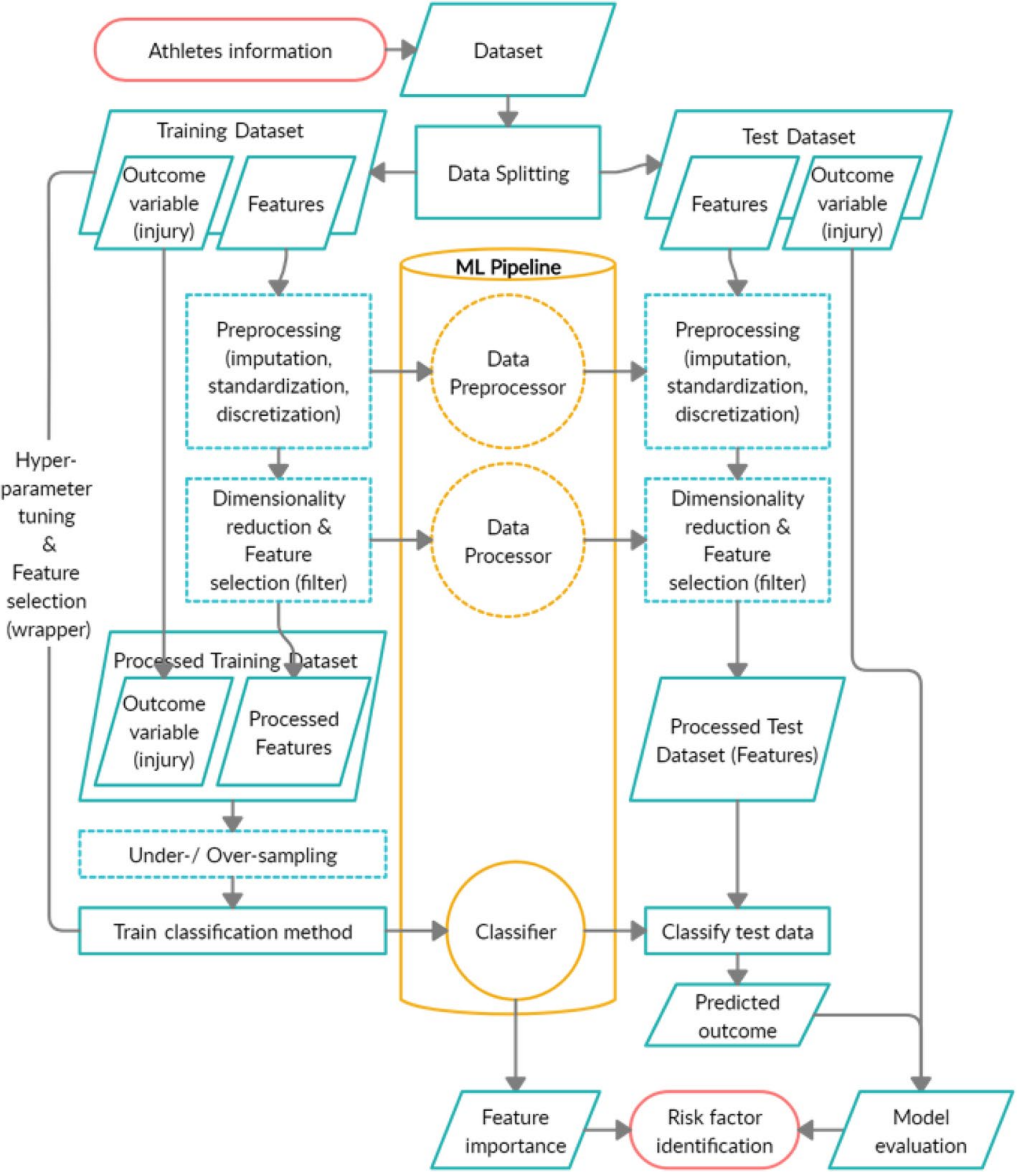
Schematic figure of the Machine Learning approach. The entire Machine Learning process is shown. Parts in dotted shapes are optional or not always necessary

AI is mostly narrowed down to Machine Learning (ML) methods although it is a very broad concept comprising every aspect of mimicking human intelligence. ML is the study of algorithms that can automatically learn from data to make new decisions [23]. Current ML methods include Neural Networks, Support Vector Machines, or Random Forests which are part of a 'Machine Learning pipeline' (Fig. 1). The available data for the ML model has to be of high quality and can be any data deemed useful for the purpose of injury prediction. This data is split in two parts (data splitting), the so-called training and test data. First, the algorithm has to learn the relationship between outcome of interest (injury or not) and the potential contributing factors (also called predictors/features/covariates/explanatory variables) from the training data set. The test data can then be used to test the prediction capacity of the algorithm learned from the training data. It is important that this quality check is not achieved on the training data, but on unseen data, hence the data splitting at the beginning. The quality and size of the data sets are important parameters for the quality of the results. To improve the quality of these large and complex datasets and to ensure optimal operation of the ML algorithms, data preprocessing methods (imputation, standardization, discretization), dimension reduction and feature selection can be applied (see Supplementary File S1). Most ML procedures further require parameter tuning, a sort of optimization of parameters which cannot be estimated directly from the data (e.g., number of trees to be used in a Random Forest). When the entire ML pipeline is fitted on the training data, the outcome of the test data is predicted. Since we know the true outcome of the test data, this allows us to evaluate our established prediction model. Finally, well-performing models provide an idea of the most important risk factors, by observing which factors have the largest influence in these models.

Considering that sport injury prediction and prevention are trending topics in sport science [12, 13, 16], the intention of this systematic review is to synthesize the evidence of sophisticated ML algorithms in sport injury prediction and prevention. Our systematic review differs from the one by Claudino et al. [12] in that we focus on injury prevention and risk factor identification together with a deeper examination of the used ML analyses. The following three topics are assessed:

1. Identify the currently used definition of ML as well as predominantly used ML methods.
2. Identify the accuracy of the currently used ML methods to predict injury.
3. Evaluate the used methods for sport injury prevention purposes.

## Methods

This systematic review was performed in accordance with the Preferred Reporting Items for Systematic Reviews and Meta-Analyses (PRISMA) guidelines [30]. The review protocol was prospectively registered at PROSPERO (International prospective register of systematic reviews—ie, CRD42020177708).

### Search strategy and inclusion/exclusion criteria

A systematic electronic search of the PubMed database was executed on March 24th 2020 to identify studies investigating Machine Learning methods in injury prediction and prevention. The following search term was used in all fields: (“deep learning” OR “artificial intelligence” OR “machine learning” OR “neural network” OR „neural networks “ OR „support vector machines “ OR „nearest neighbor “ OR „nearest neighbors “ OR „random forest “ OR „random forests “ OR „trees" OR „elastic net “ OR „ridge “ OR „lasso “ OR „boosting “ OR „predictive modeling “ OR “learning algorithms” OR „bayesian logistic regression “) AND (“sport” OR „sports “ OR “athlete” OR “athletes”) AND („injury “ OR "injuries"). We did not use limits to perform the search and no date restrictions were applied. Inclusion criteria were as follows: (i) Original studies investigating the role of machine learning for sport injury prediction and sport injury prevention, (ii) English-language studies, (iii) studies published online or in print in a peer-reviewed journal. Injury prediction had to refer to predicting either the occurrence, the severity, or the type of injuries on the basis of risk factors. The exclusion criteria were as follows: (i) not being sport-specific, (ii) not covering injury prevention or injury prediction, (iii) meeting abstracts and proceedings. Also, studies were excluded if the used approach was rather statistical than ML. This explains why, for example, two papers from Hasler et al. [19, 20] and one from Mendonça et al. [29] were not included here.

### Study selection

The titles and abstracts of all articles were screened for relevance according to the inclusion and exclusion criteria (L.M. and T.T.). If no abstract was available, the full-text article was obtained to assess the relevance of the study. The full text was subsequently reviewed for possible inclusion in the systematic review for all articles that were not excluded during the initial screening process. A third reviewer (H.E.) resolved between-reviewer discrepancies. In addition to the electronic search, the reference lists of all included articles and review articles were manually searched (C.L., T.T., H.E.) for additional relevant articles. Moreover, if any systematic reviews on ML in sport injury prediction and prevention were identified during the screening process, the reference list was screened to identify any further studies.

### Methodological quality

Methodological quality and risk of bias of included studies were determined by the Newcastle–Ottawa Scale (NOS) [45]. Eligible studies were independently rated by two authors blind to the study authors and institutions (L.M. and T.T.), with discrepancies resolved by a third author (H.E.). The NOS contains eight categories relating to methodological quality and each study was given an eventual score out of a maximum of 8 points. A score of 0–3 points equated to a low quality study, a score of 4–6 points equated to a moderate quality study, with a score of 7–8 points required for a study to be given a score of high quality.

### Data extraction

Characteristics of all included studies (i.e. participants, type of study, sample size, etc.) and about ML used (i.e. data pre-processing, classification method, etc.) were extracted independently by two reviewers (H.E. and C.L.), with a third (L.M.) resolving potential discrepancies.

### Data analysis

Two independent reviewers (L.M. and T.T.) assessed the quality of evidence using the GRADE methodology [18]. In the current review, evidence started at moderate certainty, since investigation of publication bias was not possible due to the small number of included trials. Then it was downgraded by one level for imprecision when the analysed sample was < 300 participants (serious imprecision was downgraded by two levels); and by one level for risk of bias when the mean NOS Score was < 6 out of 9. Between-reviewer discrepancies were resolved by a third investigator (R.S.).

## Results

**Fig. 2:**
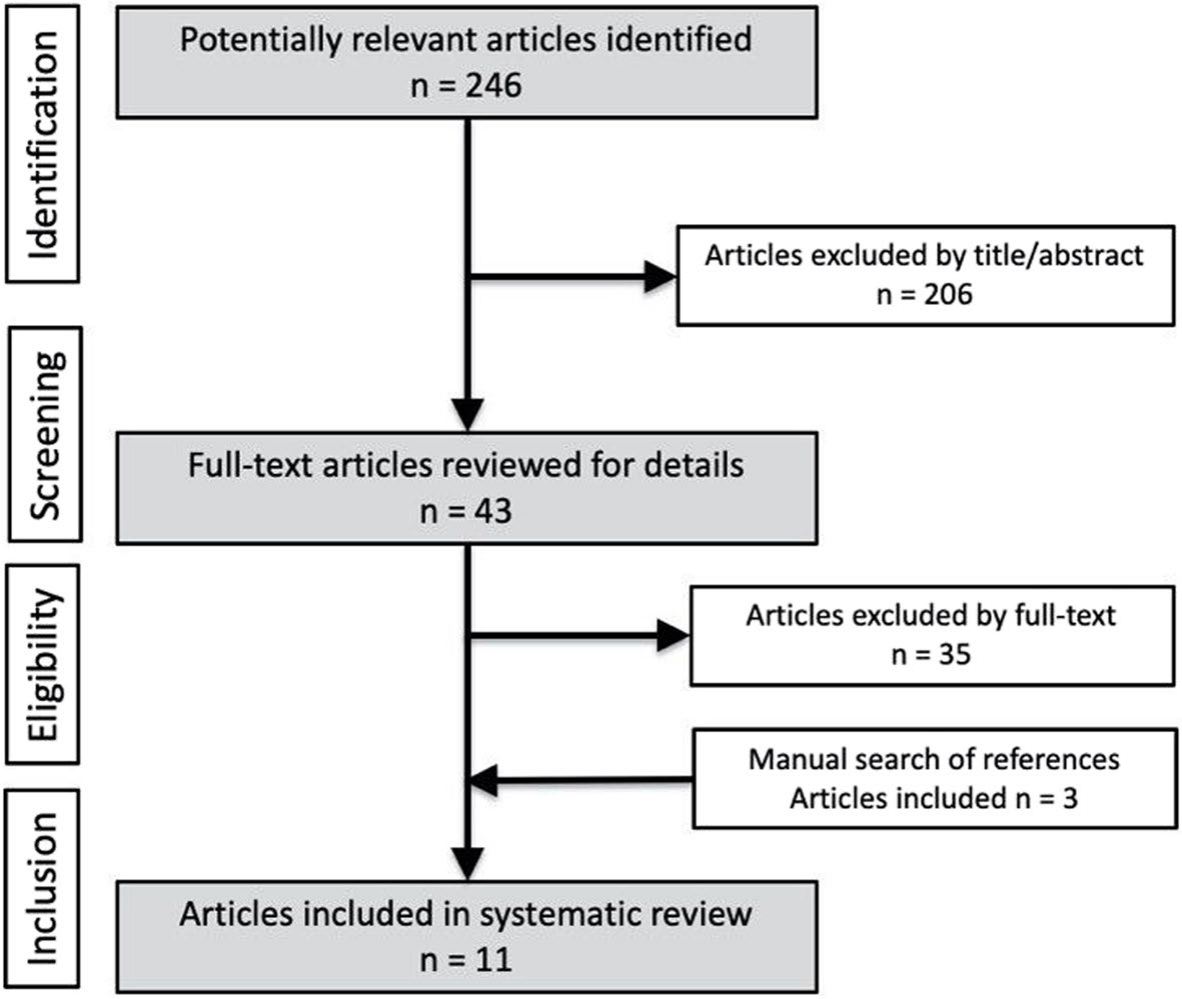
PRISMA flow chart

In the scope of this systematic literature review, 246 articles were found, and an additional three articles added by hand search from which a total of 11 articles were included according to the strict inclusion/exclusion criteria for this systematic review (Fig. 2).

### Study characteristics

Table 1 lists all details of the included studies. Studies were prospective cohort studies (*n* = 9) [2, 11, 25, 27, 32, 35, 36, 38, 41] or case–control studies (*n* = 2) [41, 46]. Most of them were performed in soccer (*n* = 4) [2, 32, 35, 36] and Australian Football (*n* = 3).[11, 27, 38] Two studies incorporated athletes from multiple sports [25, 34]. The number of participants ranged between 25 and 363. In seven studies, the athlete was the unit of observation [2, 25, 32, 34, 35, 38, 46]. In the remaining 4 studies, there were multiple observations per player [11, 27, 36, 41]. Both occurrence (*n* = 11) [2, 11, 25, 27, 32, 34–36, 38, 41, 46] and type of injury (*n* = 2) (acute/overuse [35], contact/non-contact [27]) were evaluated, whereby lower limb muscle injury was the most often assessed outcome [2, 25, 38] and only one publication investigated specifically upper limb injuries [46].

**Table 1:** Study characteristics

Authors, Year	Outcome Variable	Predictor Variables	Participants (Age Mean ± sd)	Period	Study Design	Unit of Observation	Number of Observations	Total Amount of Injuries / No. Of Injured Athletes (*N* =)^a^	Number of Features
AYALA ET AL., 2019 [2]	Occurrence of Hamstring strain injury	Individual (sport-related background, demographic, previous hamstring strain injury), psychological and neuromuscular measurements	96 Male professional soccer players from 4 teams in 1st and 2nd league in Spain 6 players that did not complete the tests and 4 players that left their teams were removed	1 season (2013–2014)	Prospective cohort	Player	86	NR/18	229
CAREY ET AL., 2018 [11]	Occurrence of non-contact injury, non-contact causing time loss injury and hamstring injury	Training load variables (+ Exponential Weighted Moving Average features and Acute Chronic Workload Ratio features)	75 male professional players from 1 team in the Australian Football League in Australia	3 seasons (2014–2016)	Prospective cohort	Player matches and player training sessions	13,867	Non-contact: 388/NR Non-contact causing time loss: 198/NR Hamstring: 72/NR	58
LÓPEZ-VALENCIANO ET AL., 2018 [25]	Occurrence of lower extremity muscle injury	Individual (sport-related background, demographic, previous injury), psychological and neuromuscular measurements	132 Male professional players in handball (34) and soccer (98) in the first three National Leagues in Spain 6 players that did not complete the tests and 4 players that left their teams were removed	1 season (2013–2014)	Prospective cohort	Player	122	32/29	151
MCCULLAGH ET AL., 2013 [27]	Occurrence of injury and injury type (contact or non-contact)	Workloads, squeeze test data, soft tissue scores, stress level, mood, sleep score, ankle flexibility, fatigue and player perceived performance, years played, player durability, age	39 male professional players from the Australian Football League in Australia	1 season (2010)	Prospective cohort	Player weeks	1210	163/NR	30
OLIVER ET AL., 2020 [32]	Occurrence of non-contact lower limb injury	Personal data (age, Body Mass Index, etc.) and neuromuscular control tests data	355 Male youth soccer players (age 14.3 ± 2.1) from Premier League and Championship clubs in England	1 season (2014–2015)	Prospective cohort	Player	355	NR/99	20
RODAS ET AL., 2019 [34]	Occurrence of Tendinopathy	Genetic markers	363 Male (89%) and female (11%) professional soccer, futsal, basketball, handball and roller hockey players (age 25 ± 6) from FC Barcelona in Spain	10 years (2008–2018)	Case–control	Player	363	199/199	1 419 369
ROMMERS ET AL., 2020 [35]	Occurrence of injury and type of injury (acute and overuse)	Anthropometric measurements, motor coordination and physical fitness	734 Male U10 to U15 youth soccer players (age 11.7 ± 1.7) of 7 premier league clubs in Belgium	1 season (2017–2018)	Prospective cohort	Player	734	NR /368	29
ROSSI ET AL., 2018 [36]	Occurrence of injury	Personal, Workload features from GPS Tracking data, previous injury	26 Male professional soccer players (age 26 ± 4) in Italy	1 season (2013–2014)	Prospective cohort	Player training session	952	23/13	55
RUDDY ET AL., 2018 [38]	Occurrence of hamstring strain injury	Age, previous hamstring strain injury, low levels of eccentric hamstring strength	362 Male professional players from the Australian Football League in Australia: 186 in 2013 (age 23.2 ± 3.6) and 176 in 2015 (age 25.0 ± 3.4)	2 seasons (2013, 2015)	Prospective cohort	Player	2013: 186 2015: 176	2013: NR/27 2015: NR/26	3 or 8
THORNTON ET AL., 2017 [41]	Occurrence of Injury	Training intensity	25 Male professional rugby players from Australian National Rugby League in Australia. Athletes were included in the dataset if they sustained more than 3 injuries in total	3 seasons (2013–2015)	Prospective cohort	Player days	NR	156/25	NR
WHITESIDE ET AL., 2016 [46]	Occurrence of ulnar collateral ligament reconstruction	Demographic and pitching performance	208 Male professional baseball pitchers from the Major League Baseball in the USA and Canada: 104 cases (age 27.3 ± 3.8) and 104 controls (age 27.8 ± 3.7)	5 years (2010–2015)	Matched Case–control	Player	208	NR/NR	14

^a^ The for the analysis relevant number is put in bold. If the unit of observation is player, then the number of injured players is relevant, since one only detects if a player gets injured at least once. If there are multiple observations per player, the total number of injuries is relevant for the analysis

### Study Quality

The methodological quality of the included studies ranged from 5 to 8 in the NOS scale (Table 2). All studies had proper ascertainment of outcome/exposure; follow-up long enough for outcomes to occur, same method of ascertainment for cases and controls; and adequacy of follow-up of cohorts/non-response rate. Seven studies (63.63%) were downgraded for methodological quality due to imprecision and three (27.27%) because of risk of bias (Table 3).

**Table 2:** Methodological quality of the included studies using the NOS scale [45]

Cohort studies	Selection	Comparability	Outcome	Conclusion
Oliver et al	****	*	***	8
Ayala et al	****		***	7
López-Valenciano et al	****		***	7
Rommers et al	****		***	7
Rossi et al	**		***	5
Carey et al	**		***	5
Ruddy et al	****		***	8
Thornton et al	**		***	5
McCullagh & Whitfort	****		***	7
Case–control studies	Selection	Comparability	Exposure	Conclusion
Rodas et al	****		***	7
Whiteside et al	****		***	7

**Table 3:** Quality of Evidence according to GRADE [18]

	Imprecision (n < 300)	Risk of bias (NOS < 6)	Conclusion
Oliver et al	N (*n* = 355)	*N* = 8	moderate-quality
Ayala et al	Y (*n* = 96)	*N* = 7	low-quality
López-Valenciano et al	Y (*n* = 132)	*N* = 7	low-quality
Rommers et al	N (*n* = 734)	*N* = 7	moderate-quality
Rossi et al	Y (*n* = 26)	*Y* = 5	very-low
Carey et al	Y (*n* = 133)	*Y* = 5	low-quality
Ruddy et al	N (*n* = 362)	*N* = 8	moderate-quality
Thornton et al	Y (*n* = 25)	*Y* = 5	very-low
McCullagh & Whitfort	Y (*n* = 39)	*N* = 7	very-low
Rodas et al	N (*n* = 363)	*N* = 7	moderate-quality
Whiteside et al	Y (*n* = 113)	*N* = 7	low-quality

### Data analysis characteristics

In all 11 papers, the outcome variable was the occurrence of injury or the type of injury, which are categorical variables, making the base models classification models. From the 11 considered papers, 9 papers used tree-based models [2, 11, 25, 32, 34–36, 38, 41], 4 papers used Support Vector Machines [11, 34, 38, 46] and 2 papers used Artificial Neural Networks [27, 38]. Eight out of 9 papers using tree-based models applied a bagging strategy [2, 11, 25, 32, 34, 36, 38, 41], whereof 5 used a Random Forest approach [11, 34, 36, 38, 41]. Four papers used boosting algorithms to construct tree ensemble methods [2, 25, 32, 35].

The training, validation and test strategy for the used ML approaches varied largely between the different studies. For the evaluation and comparison of the methods, 7 papers [2, 25, 27, 32, 34, 36, 46] used cross-validation and 4 [11, 35, 38, 41] used a single data-splitting approach. Of the former, four [2, 25, 32, 36] used stratified cross-validation, which may be especially of interest in unbalanced data, because it ensures that in both training and test set the number of positive cases (injuries) is sufficiently high. In 4 papers [11, 34, 35, 38] the training dataset was split further for tuning the hyperparameters. In 3 papers [11, 36, 37] the authors repeated their entire analysis a large number of times to adjust for the randomness in the resampling and under/oversampling methods.

Three of the discussed papers used feature selection methods [34, 36, 46]. Rodas et al. [34] used the LASSO method for selecting significant features, Rossi et al. [36] eliminated features by applying cross-validation on a separate part of the data, and Whiteside et al. [46] evaluated all possible feature subsets. Carey et al. [11] used Principal Component Analysis for reducing the dimensionality of the data instead of feature selection.

To adjust for imbalanced data, the training datasets were over-and/or undersampled in 6 papers [2, 11, 25, 32, 36, 38]. All of them used oversampling of the minority class (injuries) and 4 of them applied undersampling of the majority class (non-injuries) [2, 11, 25, 32].

Data pre-processing was used in some papers to optimize the performance of the classification methods. To solve the missing values problem, three papers [2, 25, 34] mentioned using imputation methods. In three papers [2, 25, 32], the continuous variables were transformed into categorical variables, using cut-off values found in the literature or based on the data. There was only one paper [37] that mentioned a standardization of the continuous variables.

Some of the studies had small deficits in the Machine Learning Pipeline approach. Four papers in this review had multiple observations per athlete [11, 27, 36, 41] and it seems that players may appear in both the training and test datasets, which would be a violation of the rule that the training and test dataset should be independent from each other. The results of these studies can therefore not be generalized to a bigger population. The other mistakes were made in the preprocessing phase. Four papers [2, 25, 32, 38] seemed to perform discretization or standardization on the entire dataset (including test dataset), which in that case would be an example of data leakage, i.e. using the test data in the training process. This should be avoided since it does not reflect reality as the test dataset has to be seen as future data. On the other hand, Ruddy et al. [38] independently standardized the training and test dataset. Applying different transformations on the training and test dataset will cause non-optimal operation of the classifier and can lead to lower predictive performance. A structured overview of the data analysis characteristics can be found in Table 4.

**Table 4:** Data analysis characteristics

Authors	Train, Validate and Test Strategy	Data Pre-processing	Feature Selection/ Dimensionality Reduction	Machine Learning Classification Methods	Deficits of ML Analysis
AYALA ET AL	threefold stratified cross-validation for comparison of 68 algorithms	- Data imputation: missing data were replaced by the mean values of the players in the same division - Data discretization	No	- Decision tree ensembles - Adjusted for imbalance via synthetic minority oversampling - Aggregated using bagging and boosting methods	Discretization before data splitting
CAREY ET AL	- Split in training dataset (data of 2014 and 2015) and test dataset (data of 2016) - Hyperparameter tuning via tenfold cross-validation - Each analysis repeated 50 times	NR	Principal Component Analysis	- Decision tree ensembles (Random Forests), Support Vector Machines - Adjusted for imbalance via undersampling and synthetic minority oversampling	Dependency between training and test dataset
LÓPEZ-VALENCIANO ET AL	fivefold stratified cross-validation for comparison of 68 algorithms	- Data imputation: missing data were replaced by the mean values of the players in the same division - Data discretization using literature and Weka software	No	- Decision trees ensembles - Adjusted for imbalance via synthetic minority oversampling, random oversampling, random undersampling - Aggregated using bagging and boosting methods	Discretization before data splitting
MCCULLAGH ET AL	tenfold cross-validation for testing	NR	No	Artificial Neural Networks with backpropagation	Dependency between training and test dataset
OLIVER ET AL	fivefold cross-validation for comparison of 57 models	- Data discretization using literature and Weka software	No	- Decision trees ensembles - Adjusted for imbalance via synthetic minority oversampling, random oversampling, random undersampling - Aggregated using bagging and boosting methods	Discretization before data splitting
RODAS ET AL	- Outer fivefold cross-validation for model testing - inner tenfold cross-validation for hyperparameters tuning	- Synthetic variant imputation	Least Absolute Shrinkage and Selection Operator (LASSO)	Decision tree ensembles (Random Forests), Support Vector Machines	
ROMMERS ET AL	- Split in training (80%) and test (20%) dataset - Cross-validation for tuning hyperparameters	NR	No	Decision tree ensembles - Aggregated using boosting methods	
ROSSI ET AL	- Split in dataset 1 (30%) for feature elimination and dataset 2 (70%) for training and testing - stratified two-fold cross-validation on dataset 2 - repeated 10,000 times	NR	Recursive Feature Elimination with Cross-Validation	- Decision tree ensembles - Adjusted for imbalance via adaptive synthetic sampling - Aggregated using Random Forests	Dependency between training and test dataset
RUDDY ET AL	Between Year approach: - Split in training dataset (2013) and test dataset (2015) Within Year approach: - Split in training (70%) and test (30%) dataset Both approaches: - tenfold cross-validation for hyperparameter tuning - Each analysis repeated 10,000 times	- Data standardization	No	- Single decision tree, decision tree ensembles (Random Forests), Artificial Neural Networks, Support Vector Machines - Adjusted for imbalance via synthetic minority oversampling	Standardization independent in training and test dataset
THORNTON ET AL	Split in training (70%), validation (15%), and test (15%) dataset	NR	No	Decision tree ensembles - Aggregated using Random Forests	
WHITESIDE ET AL	fivefold cross-validation for comparison of models	NR	Brute Force feature selection: Every possible subset of features is tested	Support Vector Machines	

### Performance in predicting injury occurrence

In Table 5, the study results characteristics are given for each of the included papers. For predicting the occurrence of the outcome (injury in general, muscle injury, …), seven papers used Area Under the ROC Curve (AUC) as an evaluation measure [2, 11, 25, 32, 36, 38, 41], while the remaining four papers used only metrics based on the confusion matrix, e.g. accuracy, sensitivity, specificity, precision and f1-score. Eight out of eleven studies [2, 25, 27, 32, 35, 36, 41, 46] reported appropriate to good performance of the Machine Learning prediction methods. AUC values for predicting the outcome ranged between 0.64 and 0.87, and high values were found for accuracy (75%—82.9%), sensitivity (55.6%—94.5%), specificity (74.2%—87%) and precision (50%—85%). Three papers [11, 34, 38] reported low prediction potential for their built ML models, showing low AUC (0.52—0.65) and accuracy (52%) values.

**Table 5:** Study results characteristics

Authors, Year	Performance Measures (+ Values for Best ML Model)	Predictive Performance of ML Methods	Measures of Feature Importance	Most Important Injury Predictors
Ayala et al., 2019 [2]	AUC (0.873), Sensitivity (77.8%), Specificity (83.8%)	An alternating decision tree, combined with synthetic minority oversampling and boosting gave the best results	The frequency with which each of the features appears across the tree classifiers	Sleep Quality
Carey et al., 2018 [10]	(Median) AUC (all below 0.65), Sensitivity, Specificity, Precision, False Disovery Rate, Likelihood Ratios	The proposed ML models perform only marginally better than would be expected by random chance	NR	NR
López-Valenciano et al., 2018 [25]	AUC (0.747), Sensitivity (65.5%), Specificity (79.1%)	An alternating decision tree, combined with synthetic minority oversampling and boosting gave the best results	The frequency with which each of the features appears across the tree classifiers	sport devaluation, history of muscle injury in last season
McCullagh et al., 2013 [27]	Accuracy (82.9%), Sensitivity (94.5%), Specificity (81.1%)	Indication that Artificial Neural Networks are able to derive meaningful information from the vast amount of data available to assist in the injury prediction process	NR	NR
Oliver et al., 2020 [32]	AUC (0.663), Sensitivity (55.6%), Specificity (74.2%)	The machine learning model provided improved sensitivity to predict injury	The frequency with which each of the features appears across the tree classifiers	interactions of asymmetry, knee valgus angle and body size
Rodas et al., 2019 [34]	Accuracy (52%), Sensitivity (75%), Specificity (23%)	There is low prediction potential for presence or absence of tendinopathy	The number of times that a feature (genetic predictor) received a non-zero coefficient in the LASSO analysis	rs10477683 in the fibrillin 2 gene was the most robust SNP (single-nucleotide polymorphism)
Rommers et al., 2020 [35]	F1-score (85%), Sensitivity (85%), Precision (85%)	It is possible to predict injury with high accuracy	SHAP (SHapley Additive exPlanations) summary plot	Higher predicted age at PHV (peak height velocity), longer legs, higher body height, lower body fat percentage
Rossi et al., 2018 [36]	(Mean) AUC (0.76), F1-score (64%), Sensitivity (80%), Specificity (87%) Precision (50%), Negative Predicted Value (96%)	The single Decision tree performs best in terms of precision	Mean decrease in Gini coefficient	Previous injury (exponential weighted moving average), total distance (monotony of workload feature) and high-speed running distance (exponential weighted moving average)
Ruddy et al., 2018 [38]	(Median) AUC (0.58, 0.57 and 0.52)	Eccentric hamstring strength, age, and previous hamstring strain injury (HSI) data cannot be used to identify athletes at an increased risk of HSI with any consistency	NR	NR
Thornton et al., 2017 [41]	AUC (0.74, 0.65, 0.64 and 0.64)	Machine learning techniques can appropriately monitor injury risk amongst professional team sport athletes	Number of times that each feature appears in the ensemble of decision trees	The relative importance of each training load variable varied for each player
Whiteside et al., 2016 [46]	Accuracy (75%), Sensitivity (74%), Specificity (75%), Precision (75%), False Omission Rate (26%)	Machine learning models can predict future ulnar collateral ligament surgeries with high accuracy	The frequency with which each feature appeared in the optimized models in the fivefold cross-validation	Mean days between consecutive games, pitches in repertoire, mean pitch speed, horizontal release location

### Most important injury predictors

Analysed risk factors included both modifiable (training load, psychological and neuromuscular assessment, stress level, …) and non-modifiable (demographics, genetic markers, anthropometric measurements, previous injury, …) factors (for more details, see Table 1). In 4 papers [2, 25, 32, 41], the authors have counted the number of appearances of each feature in the final ensemble of decision trees. Two papers [34, 46] counted the number of times that a feature is selected by their feature selection procedure. Rossi et al. [36] used the decrease in Gini coefficient to measure the importance of variables and Rommers et al. [35] used a SHAP summary plot [26]. This plot was based on the Shapley values in game theory and shows the importance of the variables, as well as the relation between high/low feature values and high/low injury risk. Because of the wide variety of features used over the different papers, not much consistency was found in the reported important predictors. The features that were reported twice as important were previous injury [25, 36], higher training load [36, 41], and higher body size (in youth players only) [32, 35]. Note that lower training load after previous injury might indicate a not fully recovered athlete and can hence be considered being a risk factor after previous injury [36].

## Discussion

The 11 studies included in this systematic review showed that ML methods can be successfully applied for sport injury prediction. The most promising results to predict injury risk were obtained in elite youth football players based on anthropometric, motor coordination and physical performance measures with a high accuracy of 85% [35], and in professional soccer based on a pre-season screening evaluation with a high sensitivity (77.8%) and specificity (83.8%).[2] This is in opposition with several authors who found that screening tests were not successful in predicting sports injuries [40, 43]. These results are promising in the sense that future models might help coaches, physical trainers and medical practitioners in the decision-making process for injury prevention.

Data inclusion was still limited in the analysed studies, where only selected variables were included (e.g., only anthropometric, motor coordination and physical performance measures in the study by Rommers et al. [35]). Nevertheless, the achieved accuracy was quite high and future prediction might become even higher by using smart machine learning approaches or by incorporating more data (e.g., using sensors, more intense monitoring of athletes) [44]. Future studies will need to refine the target of injury prediction with AI/ML. This can either be achieved with an increase of the number of different injuries affecting a specific population or a study cohort [35] or with a targeted inclusion of specific injuries with a high injury incidence like hamstring injuries in football or athletics [2, 38], or a high injury burden like anterior cruciate ligament (ACL) injuries in pivoting sports or ulnar collateral injuries in baseball [46]. The types and number of injury risk factors to be included in these studies are manifold and vary for each target. Large datasets may help the sports medicine community to improve the understanding of the respective influence of each factor on injury occurrence as well as their specific interactions in a given environment, allowing for a more systemic approach of sports injury prevention [6–8, 15].

In the new field of ML for sports injury prevention, the level of quality of the published studies is of utmost importance. The analysis of the methodological quality of the 11 included studies indicates that they had very-low to moderate methodological quality according to the GRADE analysis. Imprecision (i.e., a study including relatively few participants/events) is an issue that may be improved with multicentric studies. Only 3 studies [11, 36, 41] had a NOS score under 6 and only 1 study [32] scored in comparability. In fact, the main reason to a lower NOS score was lack of comparability, which indicates that either cases and controls or exposed and non-exposed individuals were not matched in the design and/or confounders were not adjusted for in the analysis. Oliver et al., the only paper considering comparability, recruited 6 professional football teams of the English Premier League and Championship and followed 355 athletes [32]. Injured and non-injured players were compared in all continuous variables and all 95% CI presented had proper range, indicating adequate matching between groups. Future studies should be aware of this common limitation and include the comparison between groups.

In terms of ML methodology, the following observations can be made from this review. (i) Tree-based models are currently the most popular ML models in sports medicine. They are easy to visualize and interpret, and they can be extended to ensemble methods for boosting and bagging purposes or adapted to be cost-sensitive. The two publications that did not use tree-based models were the first to be published on the subject [27, 46], thereby confirming the trend that more recent studies seem to adhere to this methodology. (ii) Concerning training and evaluating the ML models, a big variety between the 11 papers could be noticed. It was surprising to see that only 4 papers [11, 34, 35, 38] mentioned having tuned the hyperparameters to optimize the performance of the ML methods, since tuning hyperparameters is recommendable (though not mandatory) in order to take the most out of the ML methods. The other studies may have used values from the literature or the default values from the used software, which may have led to a failure to identify the optimal model. (iii) The findings from this review further reveal that future studies involving ML approaches in the field of sports injury prevention should aim for a higher methodological quality. One of the identified deficits of the analysed studies was the dependency between training and test datasets.

The predictive performance of the considered publications was very heterogeneous. It should be emphasized that the reported predictive performances cannot be seen as a quality measure of the ML analysis per se, because they are depending on many other factors, like the kind of included risk factors, the design of the study, the sample size or the unit of observation. This also appeared when the publications of Ayala et al. [2], Lopez-Valenciano et al. [25] and Oliver et al. [32] were compared to each other. They used similar preprocessing and processing steps and classification trees, but reported very different performance values (AUC ranging from 0.663 to 0.873). Furthermore, the reported measures (AUC, accuracy, sensitivity, specificity) might not be the best measures to evaluate the prediction models, since these measures only see black and white (injured or not injured), while probabilistic scoring rules, such as the Brier Score and the Logarithmic Loss, would be able to evaluate the exactness of a predicted probability (e.g. this player has 30% chance to get injured) as is stated by Carey et al.[10]. From a clinical point of view, it could be more informative to know the probability of injury instead of only the classification into a high or low risk profile.

When dealing with injury prediction and prevention, it is important to identify especially modifiable risk factors, which can be intrinsic or extrinsic [28]. While some studies did not provide any information on the relative importance or influence of an individual risk factor, others used the number of times that a considered variable appeared in the ensemble of decision trees. Rossi et al. [36] measured how much the predictive performance of an algorithm would decrease if a specific variable would be left out as a predictor. Rommers et al. [35] provided a visualisation of the influence of the risk factors on the predicted injury risk. Therefore, it appeared that more efforts should be done to understand the relative weight of individual risk factors on the injury risk. This approach may help guiding practitioners to apply targeted interventions to the athletes at high injury risk.

### Limitations of the systematic review

Besides investigating the outcome of machine learning algorithms in injury prediction and prevention, this systematic review also focused on the methodology of AI/ML studies, which makes some parts probably challenging to read for sports medicine clinicians. To avoid misinterpretation, a brief summary of AI/ML methods was included. It is important to stress that a previous review of Claudino et al. [12] about the use of AI in team sports provided a first overview of the topic, however it included methods that were used in a clearly statistical way, such as Bayesian logistic regression and single decision tree classifiers. Using this categorization implies that studies which were performed before the era of AI/ML and including statistical methods like linear or logistic regression would need to be considered to get a complete overview of the topic. This would seriously dilute true ML approaches. Another limitation is the fact that only PubMed database was included in this review. Even though, more relevant studies were found compared to reviews using other databases, such as e.g. in Claudino et al. [12].

## Conclusion

This systematic review showed that ML methods may be used to identify athletes at high injury risk during sport participation and that it may be helpful to identify risk factors. However, although the majority of the analysed studies did apply machine learning methods properly to predict injuries, the methodological study quality was moderate to very low. Sports injury prediction is a growing area and further developments in this promising field should be encouraged with respect to the big potential of AI/ML methods.

## Supplementary Information

**Additional file 1: S1.** Definitions of core terms important for AI application in sport injury prediction and prevention.

## Data Availability

Not applicable.
