# Supplementary material for: Machine learning methods in sport injury prediction and prevention: a systematic review
Source: J Exp Orthop. 2021 Apr 14;8:27. doi: 10.1186/s40634-021-00346-x (PMC8046881; doi:10.1186/s40634-021-00346-x)
Supplement: Supplementary file 1 — Additional file 1: S1. Definitions of core terms important for AI application in sport injury prediction and prevention. [file 40634_2021_346_MOESM1_ESM.docx]

S1: Definitions of core terms important for AI application in sport injury prediction and prevention.

| **General Data Analysis concepts** |  |
| --- | --- |
| Regression analysis | Analysis of the relation between a continuous outcome variable and one or more explanatory variables (also called features/predictors/covariates) |
| Classification analysis | Analysis of the relation between a categorical outcome variable and one or more explanatory variables (also called features/predictors/covariates) |
| **Machine Learning Concepts** |  |
| Data splitting | Separation of the data into training set and test/validation set |
| Cross-validation | Data splitting technique (either of the entire dataset to evaluate the performance of a ML procedure or of the training set e.g. to tune hyperparameters) where each observation once serves for evaluation of a ML method and for the rest for its training |
| Feature selection | Reduction of the number of features by keeping only those that really contribute to the outcome |
| Hyperparameters | Model parameters that are not automatically learned from the data and so should be provided before fitting the model |
| Over- and undersampling | Sets of techniques to either reduce the majority class or blow up the minority class |
| Ensemble learning | Combination of individual (weak) ML techniques to yield a more precise prediction (aim is to reduce bias and avoid overfitting) |
| Bootstrapping | Sampling technique where new data sets are created by drawing with replacement from the original data set |
| Bagging | Short for “bootstrap aggregating”, an ensemble learning technique that aggregates the predictions of several bootstrapped data sets |
| Cost-sensitive | Type of learning that takes into account some cost of misclassification |
| Boosting | An ensemble learning technique that trains weak learners sequentially, each learner correcting its predecessor |
| **ML classification & regression methods** |  |
| Decision trees | A (single-)tree-shaped classification or regression analysis |
| Artificial Neural Networks | Family of ML methods that mimic the biological neural network |
| Support Vector Machines | Family of ML methods that construct separating hyperplanes mainly for classification purposes (can also be used for regression) |
| Random Forest | Bagging technique based on decision trees |
